# Three new genera of Neotropical Mimallonidae (Lepidoptera, Mimallonoidea, Mimallonidae) with descriptions of three new species

**DOI:** 10.3897/zookeys.566.7344

**Published:** 2016-02-18

**Authors:** Ryan A. St. Laurent, Carlos G. C. Mielke

**Affiliations:** 1 Cornell University, Comstock Hall, Department of Entomology, Ithaca, NY 14853-2601 USA; 2 Caixa Postal 1206, 84.145-000 Carambeí, Paraná, Brazil

**Keywords:** Auroriana, biodiversity, Cicinnus, Micrallo, Neotropical, taxonomy, Tostallo

## Abstract

Three new genera of Mimallonidae are described. The monotypic genus *Tostallo* **gen. n.** is erected to contain “*Perophora*” *albescens* Jones, 1912, which was previously placed in the preoccupied genus *Perophora* Harris, 1841 and was never formally moved to a valid genus. *Perophora* is a junior homonym of *Cicinnus* Blanchard, 1852, but the name *albescens* is not appropriately placed in *Cicinnus* due to external and genitalia characteristics entirely unique to the species *albescens*. The female of *Tostallo albescens* **comb. n.** is described and both sexes are figured for the first time. *Auroriana* **gen. n.** is erected to contain *Auroriana florianensis* (Herbin, 2012), **comb. n.** previously described as *Cicinnus florianensis*, and two new species: *Auroriana colombiana* **sp. n.** from Colombia and *Auroriana gemma* **sp. n.** from southeastern and southern Brazil. The female of *Auroriana florianensis* is described and figured for the first time. Finally, the monotypic genus *Micrallo* **gen. n.** is erected to include a new species, *Micrallo minutus* **sp. n.** described from northeastern Brazil.

## Introduction

Schaus (1928) described 16 of the 28 currently recognized Mimallonidae genera, with only *Naniteta* Franclemont, 1973 and *Arianula* Herbin, 2012 having been described since then. Franclemont (1973) recognized strong affinities between all of the genera and speculated that the family must be recently diversified. The number of genera in Mimallonidae is high relative to the small size of the family, wherein only a little more than 200 species have been described (Becker 1996, Herbin 2012, 2015, Herbin and Mielke 2014, Herbin and Monzón 2015, St Laurent and Dombroskie 2015). Therefore, many of the genera are represented by three or fewer species (St Laurent and Dombroskie 2015), including the three new genera described here.

Currently there is no accepted subfamily arrangement for the family Mimallonidae. Schaus (1928) proposed two subfamilies based primarily on the presence/absence of the frenulum. Schaus frequently failed to recognize the presence of vestigial frenula when they were in fact present, hence his subfamily arrangement has been rejected by most contemporary authors (Pearson 1951, 1984, Franclemont 1973, Herbin 2012, St Laurent and Dombroskie 2015, but see Becker 1996 and Herbin and Monzón 2015). We continue to recognize the lack of a clear subfamily arrangement awaiting a higher-level treatment of the family.

Recent interest in the taxonomy of Mimallonidae has resulted in numerous new species (Herbin 2012, 2015, Herbin and Mielke 2014, Herbin and Monzón 2015, St Laurent and Dombroskie 2015) described over a short period of time, displaying the previous lack of work on the group. Recent efforts by Herbin (2012) and on-going investigations by the first author of the present article have resulted in new genera, three of which are described herein.

## Methods

Dissections were performed as in Lafontaine (2004). Morphological, including genitalia, terminology follows Kristensen (2003). Figures were manipulated with Adobe Photoshop CS4 (Adobe 2008).

Male genitalia are figured in natural color with CS4 “auto color” used to improve white backgrounds. Most genitalia were photographed with a Macroscopic Solutions Macropod Pro and Canon EOS 6D DSLR camera body using the Macro Photo MP-E 65mm f/2.8 1–5× Manual Focus Lens for EOS except when received from other individuals or institutions. Thirty (3×) photographs were taken of each specimen in ethanol under glass, and stacked using Zerene Stacking Software.

Maps were created with SimpleMappr (Shorthouse 2010) and edited with CS4. All geographical co-ordinates are approximate, and are based on the localities provided on specimen labels. Co-ordinate data were acquired with Google Earth (2015).

Specimens from the following collections were examined:

CGCM
 Coll. Carlos G. C. Mielke, Curitiba, Paraná, Brazil 


COM
 Coll. Olaf H. H. Mielke, Curitiba, Paraná, Brazil 


CPC
 Coll. Philippe Collet, Caen, France 


DZUP
 Coll. Pe. Jesus S. Moure, Departamento de Zoologia, Universidade Federal do Paraná, Curitiba, Paraná, Brazil 


MNHN
Muséum nationale d’Histoire naturelle de Paris, France 


MZSP
 Coll. Museu de Zoologia, Universidade de São Paulo, São Paulo, São Paulo, Brazil 


BMNH
 The Natural History Museum [statutorily British Museum (Natural History)], London, U.K. 


RAS
 Research collection of Ryan A. St. Laurent, Ithaca, New York, USA 


USNM
 National Museum of Natural History [formerly United States National Museum], Washington D.C., USA 


## Results and discussion

### Tostallo

Taxon classificationAnimaliaLepidopteraMimallonidae

St Laurent & C. Mielke gen. n.

http://zoobank.org/5C06B802-4FA9-4DDA-B6E6-B4D422292E0E

#### Type species.

*Perophora albescens* Jones, 1912.

#### Etymology.

The genus name is derived from the toasted (*tostus* Latin) appearance of the white forewings, which is unique among Mimallonidae and reminiscent of toasted marshmallows; + the ending –llo, which is shared with *Mimallo* Hübner, [1820], the type genus of Mimallonidae. The genus name is masculine.

#### Diagnosis.

The monotypic *Tostallo* is remarkable among Mimallonidae in that the ground color is nearly white and the forewings rounded, a combination of characters seen nowhere else in the family. Male genitalia are unique in that the gnathos is formed by two large columnar structures with multiple invaginations and internal wrinkles. Female genitalia can be recognized by the wrinkled, setae covered accessory part of segment VIII, which encircles the papillae anales, as well as by the apophyses posteriores, which are hollow, almost balloon-like lobes, rather than thin rods as in other genera.

#### Description.

**Male.** *Head*: Width half that of thorax, light brown, eyes bordered posteriorly by dark-brown scales encircling head, forming dark mane; palpus not extending beyond frons, covered dorsally in darker brown scales; antenna opaque yellow, bipectinate to tip, rami increasing in length from antennal base to roughly middle of antennal length where rami length continuously decrease until terminus. *Thorax*: Pale tan brown, lighter near wing base, darker mesally, overall lightly speckled with darker petiolate scales. *Legs*: Color as for thorax, but tarsus light brown, petiolate scales present. Tibial spurs very thin, elongate, clothed in fine white scales. *Forewing dorsum*: Forewing length: 12–13 mm, avg. 12.5 mm, Wingspan: 23–26.5, n = 3. Subtriangular, outer margin convex, apex not pronounced. Ground color cream to white, overall lightly speckled by dark petiolate scales; outer margin fringed, grey. Antemedial line faint pale brown, somewhat wavy, especially near costa. Postmedial line dark brown, waxy, convex and mostly evident from costa to Rs3. Basal and medial areas proximately concolorous with thorax; medial area distally gray reaching inner margin towards wing base. Submarginal band darker, compressed, sometimes absent between postmedial line and marginal band. The latter grayish brown, delineated by an undulating line suffused with white distally. Discal spot marked by small, tan, crescent-shaped mark. Black patch of scales at apex extends to Rs3. *Forewing ventrum*: Similar to dorsum but more yellow brown, white and gray suffusions absent, submarginal and marginal bands lighter, postmedial line continuous, submarginal and antemedial lines absent. *Hindwing dorsum*: Coloration as for forewing dorsum, but lighter. Antemedial line brown, partially visible near inner margin. Postmedial line faintly marked, bordered by white distally. Submarginal band grayish brown. Marginal band lighter. *Hindwing ventrum*: Follows same pattern as forewing ventrum but discal mark absent. Frenulum a single, relatively-thick bristle. *Venation*: Typical of Mimallonidae, very similar to *Cicinnus melsheimeri* (Harris, 1841), but forewing M_1_ and M_2_ originate from cell nearer to R and CuA_1_ respectively, than to each other. *Abdomen*: Short, not exceeding hindwing tornus, stout, coloration as for thorax, ventrally pale. *Genitalia*: (Fig. 4) n = 4. Simple; tegumen reduced to slender rod. Vinculum box-like, not projected anteriorly. Uncus simple, subtriangular, truncated apically. Gnathos as two unfused, heavily-sclerotized, multiply-invaginated columnar structures with multiple internal folds. Valves short, irregularly shaped, somewhat triangular, truncated apically forming lobe, sclerotization weak mesally with heavily sclerotized fingerlike projection subapically. Juxta partially fused to phallus, separate portion of juxtal plate embedded in anellus. Phallus short, pistol shaped; flattened, open dorsally; base of phallus with short, anteriorly-curved lobe, vesica small, somewhat bag-like. **Female.** *Head*: As for male. *Thorax*: As for male. *Legs*: As for male, but tibial spurs slightly longer. *Forewing dorsum*: Forewing length: 14–16.5 mm, avg. 15.25 mm, Wingspan: 26–35 mm, n = 2. As for male but slightly longer, black patch of scales at forewing apex extended to Rs4 rather than Rs3. *Forewing ventrum*: Similar to dorsum but yellower brown. Antemedial line absent. Postmedial line continuous, submarginal band lighter. *Hindwing dorsum*: As for male but somewhat broader. *Hindwing ventrum*: Follows same pattern as forewing ventrum but discal mark absent. Frenulum apparently absent or highly reduced. *Abdomen*: As in male but more robust, sclerotized bands present ventrally on segment VIII. *Genitalia*: (Figs 5, 6) n = 2. Papillae anales setose, somewhat flattened ventrally, covered in short denticles from which very short setae originate. Apophyses anteriores very short, robust, flattened; apophyses posteriores as hollow, rounded, balloon-like lobes. Ductus bursae relatively long, corpus bursae bag-like with slight sclerotization terminally. Dorsal sclerotization of tergite VIII thin, folded inward, arc-like, with asymmetrical ridges on lateral sides of central arc. VIII encircles genitalia as thick, moderately sclerotized, setae covered, finely wrinkled structure, invaginated around entire circumference. Ostium unsclerotized. Lamella antevaginalis reduced, subtriangular, small notch apically.

**Figures 1–3.**
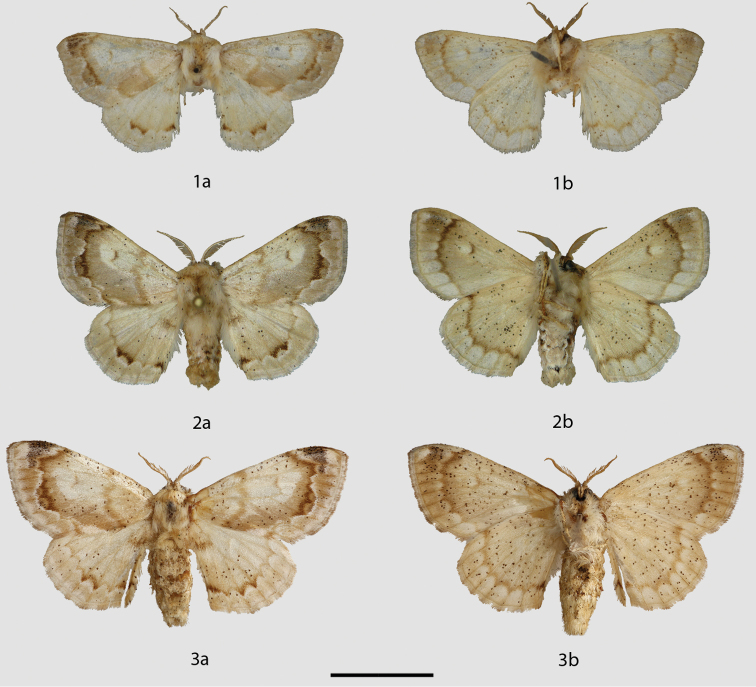
*Tostallo albescens* adults, **a** recto, **b** verso. **1** Holotype ♂, Brazil, São Paulo (BMNH) **2** ♂, Brazil, São Paulo, Guapiara, Paivinha, 800 m (CGCM) **3** ♀, Brazil, Rio Grande do Sul (BMNH). Scale bar = 1 cm.

**Figure 4.**
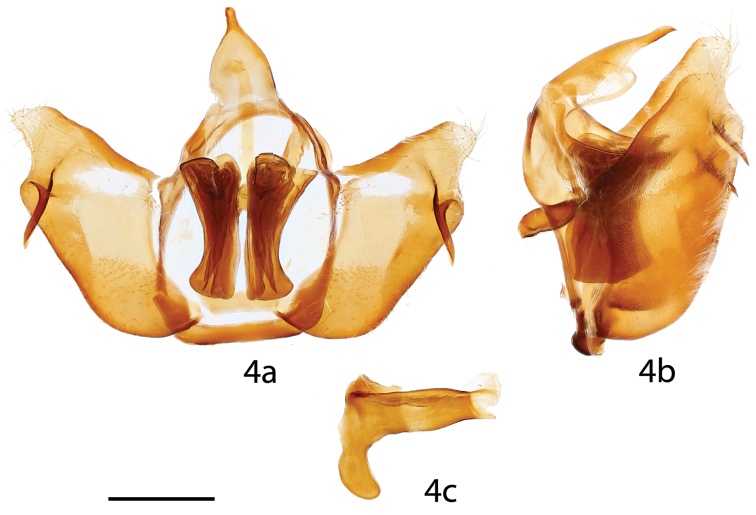
*Tostallo albescens* male genitalia, **a** ventral, **b** lateral, **c** phallus. Brazil, São Paulo, Alto da Serra [St. Laurent diss.: 10-21-15:4] (BMNH). Scale bar = 1 mm.

**Figures 5, 6.**
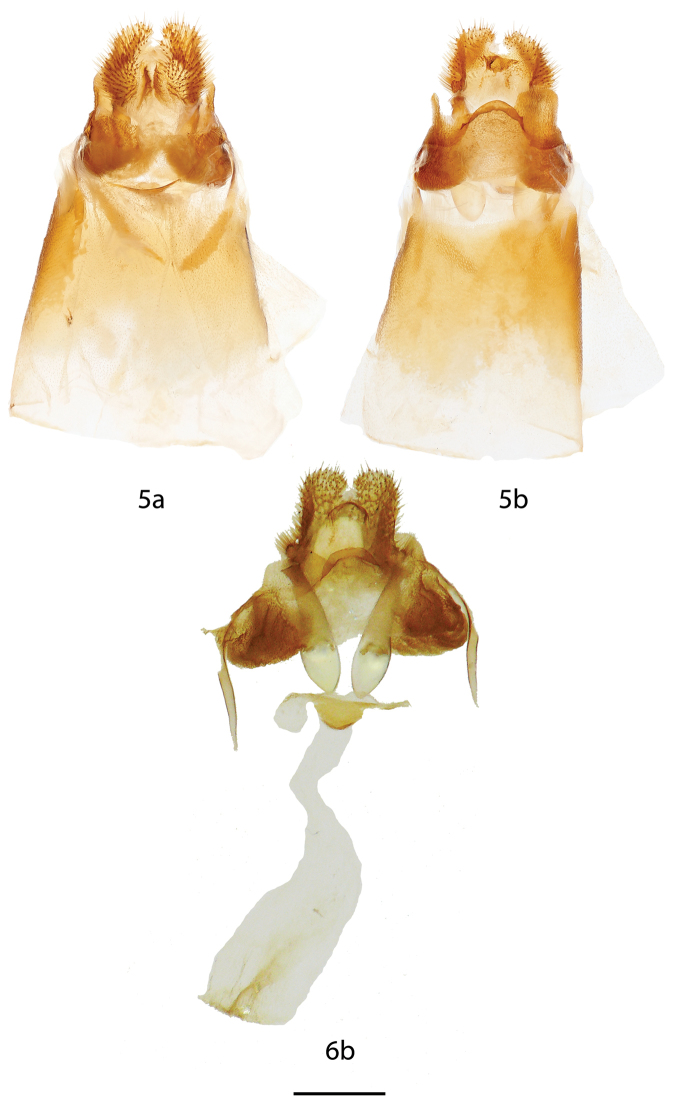
*Tostallo albescens* female genitalia, **a** ventral, **b** dorsal. **5** Brazil, Rio Grande do Sul [St. Laurent diss.: 10-21-15:6] (BMNH) **6** Brazil, Paraná, Curitiba [C. Mielke diss.: 51.381] (COM). Scale bar = 1 mm.

#### Remarks.

*Perophora albescens* Jones, 1912 was described in the preoccupied genus *Perophora* Harris, 1841 (*Perophora* Wiegmann, 1835 [Tunicata]) despite the fact that another name, *Ptochopsyche* Grote, 1896, was proposed as a replacement well before Jones’s description. Both *Perophora* and *Ptochopsyche* are currently treated as synonyms of *Cicinnus* Blanchard, 1852 (Becker 1996). However, *Tostallo albescens* comb. n. lacks the external and genital characters that are “characteristic” of the poorly defined genus *Cicinnus* (sensu Franclemont 1973, Herbin 2012, 2015, Herbin and Mielke 2014, Herbin and Monzón 2015), namely in that the forewings are not falcate and the valves of the male genitalia are simple rather than variously reduced as in *Cicinnus melsheimeri*, the type species of both *Perophora* and *Ptochopsyche*. Thus it is not appropriate to merely consider *albescens* within *Cicinnus*.

Schaus (1928), the last to completely revise the family, was unable to “identify” “*Perophora albescens*” and only re-iterated Jones’s description without assigning the species to a valid genus. Additionally, Becker (1996) did not list this species. Further compounding the ambiguity of the taxon *albescens*, neither sex of this species has been previously figured. While the validity of the species has never been questioned, Schaus’s inability to locate the holotype likely has led to this taxon being ignored or overlooked in all subsequent literature. Given the opportunity to re-examine Jones’s original specimens, including the holotype, and perform comparisons to all known Mimallonidae genera, it is readily apparent that *Tostallo albescens* comb. n. is unique among Mimallonidae and should be transferred to a new, monotypic genus. We therefore make this long overdue change in the present work, moving the name *albescens* from the preoccupied *Perophora* to a new genus, to solidify its uniqueness in the family. We provide figures of the holotype male, an additional male and female, as well as the genitalia of both sexes to allow for better recognition of this rarely reported enigmatic species.

### Tostallo albescens

Taxon classificationAnimaliaLepidopteraMimallonidae

(Jones, 1912) comb. n.

**Figure 7.**
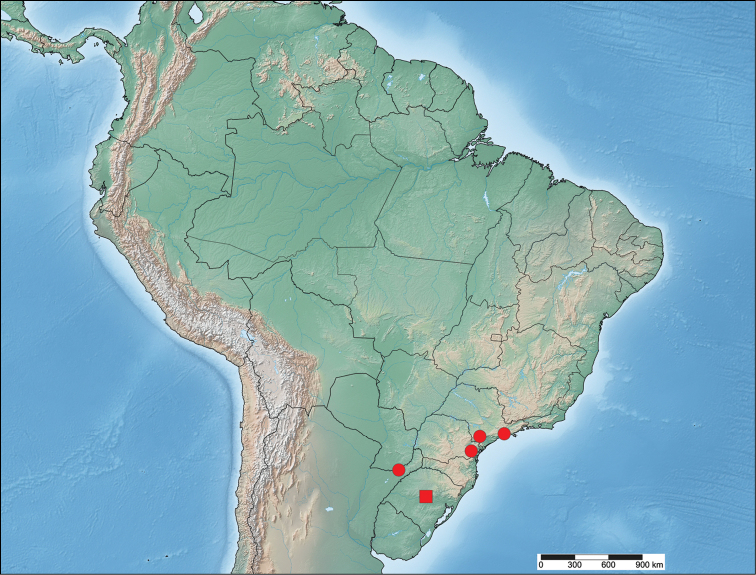
Distribution of *Tostallo albescens*. The square represents a generalized (center of the state) locality for Rio Grande do Sul, a state for which detailed locality data is lacking.

Figs 1–3 , 4 , 5, 6 , 7

Perophora albescens Jones, 1912: 435
Perophora albescens Jones; Schaus 1928: 671–672

#### Type material.

**Holotype**, ♂: S. Paulo, S. E. Brazil./ Perophora albescens, Type ♂ .D. Jones/ E. D. Jones Coll., Brit. Mus. 1919–295/ BMNH(E) #805419/ Mimallonidae BMNH(E) Slide #001/ (BMNH, examined). Type locality: Brazil: São Paulo.

#### Additional specimens examined.

(5 ♂, 2 ♀) **ARGENTINA: Misiones**: 1 ♂, Posadas: 26.IX.1921, BMNH(E) 1378118, St. Laurent diss.: 10-21-15:5 (BMNH). **BRAZIL: Paraná**: 1 ♂, 1 ♀, Tirol das Torres, Uberaba, Curitiba, 900 m: 2.III.2000, 2.I.2001, O. Mielke leg., C. Mielke diss.: 51.381, 53.261 (COM 51.381, 53.261). **Rio Grande do Sul**: 1 ♀, illegible collector and date: Joicey Coll. Brit. Mus. 1925–157, BMNH(E) 1378120, St. Laurent diss.: 10-21-15:6 (BMNH). **São Paulo**: 1 ♂, Guapiara, Paivinha, 800 m: 16–19.IX.2005, C. Mielke leg., (CGCM 28.855). 1 ♂, Alto da Serra: IX.1935, Coll. R. Spitz, Brit. Mus-112(?), BMNH(E) 1378119, St. Laurent diss.: 10-21-15:4 (BMNH). 1 ♂, “S.E. São Paulo, 750 m”: E.D. Jones, E.D. Jones Coll. Brit. Mus. 1919–295, BMNH(E) 1378117 (BMNH).

#### Diagnosis.

See genus diagnosis.

#### Description.

See genus description.

#### Distribution

(Fig. 7). *Tostallo albescens* primarily inhabits the Brazilian Atlantic Forest in the states of São Paulo and Paraná. A single record from Rio Grande do Sul lacks more specific data, thus we are unable to determine if this record comes from the Atlantic Forest region in the northeastern part of the state or from elsewhere. A single record from Misiones, Argentina, suggests that this species is more widespread in the inland ecoregions of the Atlantic Forest biome.

#### Remarks.

As mentioned in the genus remarks above, this species is unique among Mimallonidae, namely because of the white ground color and rounded forewings. This species is poorly represented in collections.

### Auroriana

Taxon classificationAnimaliaLepidopteraMimallonidae

St Laurent & C. Mielke gen. n.

http://zoobank.org/31C8B6C2-3B3D-4CF4-94EB-BBC312041FDF

#### Type species.

*Auroriana colombiana* St Laurent & C. Mielke, sp. n.

#### Etymology.

This genus is named for the sunrise (*aurora* Latin) because of the pink and orange coloration of all species in the genus. These colors are reminiscent of the light scattering phenomenon of the rising sun. The genus name is feminine.

#### Diagnosis.

*Auroriana* can be distinguished from all currently described Mimallonidae by the orange and diffuse pink coloration on all wings, and by the pink and beige head and thorax. Male genitalia are unique in the combination of the following characters: variably-shaped, heavily-sclerotized, unfused gnathos processes, relatively-simple valves, and downward-curved phallus with a distinct lobe-like process at the base. Only a few other similarly-sized, orange and pink Mimallonidae species are known: *Druentica fatella* (Schaus, 1905), *Trogoptera semililacea* (Dognin, 1916), and the species of *Reinmara* Schaus, 1928. These species, while superficially similar to *Auroriana*, can be easily differentiated by the straight forewing postmedial line and by the stark contrast between the completely or nearly completely pink medial region and the orange postmedial region rather than the much more diffuse pink coloration in *Auroriana*. Additionally, the dark brown wing fringes accent the notch at the tornus of both *Druentica fatella* and *Trogoptera semilacea*, a character not seen in *Auroriana*. *Druentica fatella* has a characteristic dark tuft of scales at the terminus of the abdomen, which is absent in *Auroriana*. Furthermore, the genitalia of these species are entirely distinct from those of *Auroriana*, where all major characters pertinent to the diagnosis of *Auroriana* are absent. Interestingly, the present study reveals that *Druentica fatella* likely does not belong to its present genus due to a combination of external and genitalia characters unlike what is seen in other species of *Druentica*.

#### Description.

**Male.** *Head*: Width roughly two thirds that of thorax, pinkish; labial palpus not extending beyond frons, pink; antenna bipectinate to terminus; rami increasing in length from antennal base to roughly one fifth of antennal length where rami length is nearly constant for following fifth of antennal length after which rami continuously decrease in length until terminus. *Thorax*: Pink and beige, thickly covered in long scales, darker petiolate scales absent. *Legs*: Light pink, vestiture long, tarsus usually tan, tibial spurs similar to those of *Eadmuna* Schaus, 1928. *Forewing dorsum*: Forewing length: 16–21 mm. Somewhat triangular, slight inward notch at tornus, margin convex mesally. Ground color light orange brown, overall very sparsely speckled by dark petiolate scales. Antemedial line faint pink, nearly straight or bowed slightly. Postmedial line slightly or moderately bowed inward, dark brown, outward edge lined with very pale pink band of varying width, postmedial line angled sharply towards costa after passing Rs4. Discal spot a small black mark. Antemedial area usually more solid pink than diffusely shaded medial area. Sub- and marginal areas darker than medial area. *Forewing ventrum*: As for forewing dorsum but antemedial and postmedial lines faint or absent; pink suffuse line near apical quarter extending from costa to postmedial suffusion. *Hindwing dorsum*: Coloration as for forewing dorsum, but less pink, postmedial line may lack outer pink edging. *Hindwing ventrum*: As in for forewing ventrum but postmedial line better developed, lobed outward mesally. Frenulum a single bristle. *Venation*: Typical of Mimallonidae, very similar to *Cicinnus melsheimeri* but CuA_1_ bent more posteriorly. *Abdomen*: Short, not extending beyond hindwing tornus, stout, coloration as for thorax but with more beige than pink. *Genitalia*: Somewhat complex; tegumen very broad to narrow, subtriangular, or more rectangular. Vinculum ovoid or irregularly shaped, lobes present mesally below gnathos, or when not present, elongated, trumpet-like structures present at base of valves instead; uncus tubular or triangular, in most species hardly differentiated from tegumen; gnathos as two unfused heavily-sclerotized processes, either roughly triangular or two-pronged. Valves triangular, with triangular or rounded saccular edge lobe, valve with mesal tooth present in one species; saccular edge of valve with longer, heavier setae in Amazonian species. Juxta fused to phallus, with elongate ventral process connecting phallus at base of valves. Phallus variable in length, but always curved downward; basally with ventrally angled elongation, terminus with hook-like process or simple. **Female.** *Head*: As for male, but antennal rami shorter. *Thorax*: As for male, but with more beige scales. *Legs*: As for male. *Forewing dorsum*: Forewing length: 17 mm. As for male, but broader, pink suffusions reduced, ground color more olive green than orange. *Forewing ventrum*: As for forewing dorsum but pinkish suffusions nearly absent, postmedial line absent, discal mark more pronounced. *Hindwing dorsum*: As for male but slightly broader, dark petiolate scales more numerous, especially antemedially. *Hindwing ventrum*: Following same pattern as forewing ventrum. *Abdomen*: As for male, but broader. Sternite VIII with pair of short sclerotized bands posteriorly. *Genitalia*: Papillae anales broad, somewhat rectangular, covered in setae, increasing in length at base. Apophyses anteriores with curved tips; apophyses posteriores robust, wide, slightly longer and broader than apophyses anteriores. Ductus bursae very long, somewhat sclerotized near ostium, ductus not differentiated from long, narrow corpus bursae. Dorsal sclerotization of tergite VIII as narrow band with posteriorly directed mesal arc with membranous center. Lamella antevaginalis very large, broad, trapezoidal, notched mesally.

#### Remarks.

This new genus is erected to include three South American species. All three species are known from very few specimens, being poorly represented in collections. Herbin (2012) was the first to recognize the uniqueness of *Auroriana florianensis* at the time of its original description, but did not describe a new genus in which to place it. Upon the discovery of two additional species similar to *Auroriana florianensis*, the first author determined that external morphology and male genital characteristics united these three species and set them apart from all other described Mimallonidae genera. We hereby describe a new genus in which to place the previously described *Auroriana florianensis* and the two new species described below.

#### Key to species of Auroriana*

**Table:** 

1	Forewing postmedial line nearly straight (Figs 8, 12)	**2**
–	Forewing postmedial line bowed inward toward thorax (Figs 9–11)	***Auroriana florianensis***
2	Hindwing postmedial line thin, well defined, pale diffuse outer band absent	***Auroriana colombiana***
–	Hindwing postmedial line as pale, pink, diffuse band	***Auroriana gemma***

*Note: the females of *Auroriana colombiana* and *Auroriana gemma* are unknown.

**Figures 8–12.**
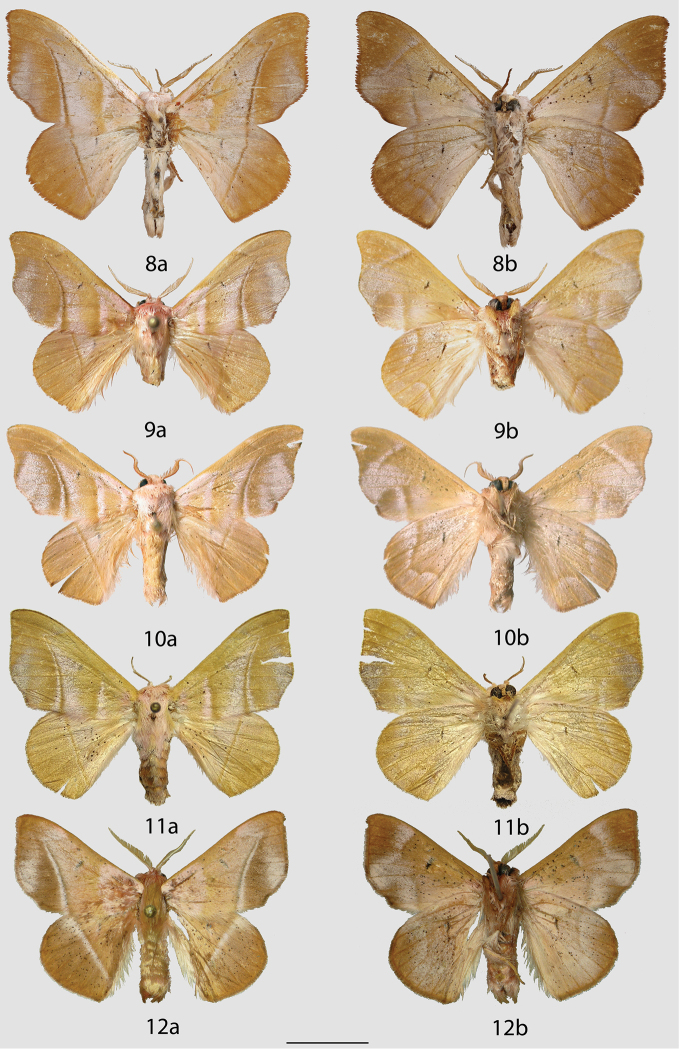
*Auroriana* adults, **a** recto, **b** verso. **8** *Auroriana colombiana* holotype ♂, Colombia, Meta, Río Negro, 800 m (BMNH) **9** *Auroriana florianensis* holotype ♂, French Guiana, Piste du Dégrad Florian, PK 12 [photo courtesy of MNHN] (MNHN) **10** *Auroriana florianensis* ♂, French Guiana, St. Jean du Maroni, Plateau des Mines, PK 1.3 [photo courtesy of MNHN] (MNHN) **11** *Auroriana florianensis* ♀, French Guiana, Réserve des Nouragues, Inselberg [photo courtesy of CPC] (MNHN) **12** *Auroriana gemma* holotype ♂, Brazil, Santa Catarina, São Bento do Sul, Rio Natal, 550 m (DZUP). Scale bar = 1 cm.

### Auroriana colombiana

Taxon classificationAnimaliaLepidopteraMimallonidae

St Laurent & C. Mielke sp. n.

http://zoobank.org/3BDB9C32-B1A1-43F1-A5D5-EE3815262CE3

**Figures 13, 14.**
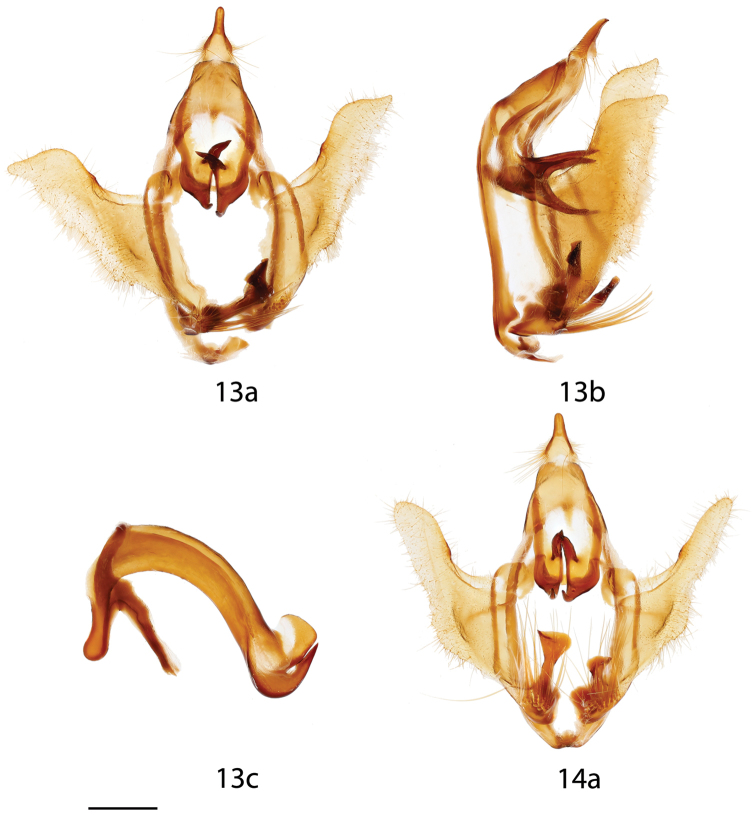
*Auroriana colombiana* male genitalia, **a** ventral, **b** lateral, **c** phallus. **13** Holotype, Colombia, Meta, Río Negro, 800 m [St. Laurent diss.: 11-3-15:1] (BMNH) **14** Paratype, Colombia, Meta, Río Negro, 800 m [St. Laurent diss.: 10-21-15:3] (BMNH). Scale bar = 1 mm.

Figs 8 , 13 , 14 , 19

#### Type material.

**Holotype**, ♂: Ob. Rio Negro [Upper Río Negro], Ost Colombia 800 m, Coll. Fassl/ Joicey Coll., Brit. Mus. 1925–157/ BMNH(E) 1378121/ St. Laurent diss.: 11-3-15:1/ HOLOTYPE male *Auroriana colombiana* St Laurent and C. Mielke, 2016 [handwritten red label]/ (BMNH). Type locality: Colombia: Meta: Rio Negro.

**Paratype**, 1 ♂: **COLOMBIA**: Upper Río Negro [Meta], 800 m: Coll. Fassl, Joicey Coll., Brit. Mus. 1925–57, BMNH(E) 1378116, St. Laurent diss.: 10-21-15:3 (BMNH). Paratype with the following yellow label: PARATYPE male *Auroriana colombiana* St Laurent and C. Mielke, 2016.

#### Diagnosis.

This new species is most similar to *Auroriana florianensis* in adult habitus but can easily be differentiated by the larger size, darker, straighter, better defined postmedial lines on all wings, and the more solid pink medial area. The genitalia also immediately distinguish these two species: in *Auroriana colombiana* the valves are narrower and less triangular, and the paired processes of the gnathos are two-pronged structures, rather than thick, triangular, basally-spined structures as in *Auroriana florianensis*. The phallus is similar in both species, but in *Auroriana colombiana* it is larger, more deeply curved, and the distal curve is more developed and backwardly angled.

#### Description.

**Male.** *Head*: Light pink, eyes bordered posteriorly by black scales; antenna opaque yellow; labial palpus small, dorsally with darker scales. *Thorax*: Pink, interspersed with beige scales. *Legs*: Light pink, vestiture long, tarsus pale brown, tarsal spurs relatively long, clothed in pink scales except naked, finely-pointed apical tip. *Forewing dorsum*: Forewing length: 20–21 mm, avg. 20.5 mm, Wingspan: 36–38 mm, avg. 37 mm, n = 2. Triangular, slight inward notch at tornus, margin convex mesally. Ground color light orange brown, medial region very sparsely speckled by dark petiolate scales. Antemedial line diffuse, faint pink, nearly straight. Postmedial line bowed inward slightly, dark brown, outward edge finely lined with pale pink, especially near tornus, postmedial line angled sharply towards costa after passing Rs4. Antemedial area pink. Postmedial area darker brown orange with pale pink-gray suffusion near wing margin, medial area lighter orange with pink suffusion, especially along postmedial line. Discal spot a small black, elongated mark. *Forewing ventrum*: Similar to dorsum but postmedial line very faint; antemedial line absent; deeper orange overall with reduced pink suffusion. *Hindwing dorsum*: Coloration as for forewing dorsum; well-defined postmedial line lacks outer pink edging. *Hindwing ventrum*: Follows same pattern as forewing ventrum but pinker, lighter overall. *Abdomen*: As for genus, coloration as for thorax, but less pink. *Genitalia*: (Figs 13, 14) n = 2. Tegumen narrow, rectangular. Vinculum ovoid with small pointed projection ventrally between saccular bases of valves. Uncus small, triangular. Gnathos as two unfused heavily-sclerotized, two-pronged processes. Valves simple, bent upward, saccular edge with mesal lobe, base of valve with elongated, trumpet-shaped processes, process of left valve longer than that of right; base of valves with long, thick deciduous setae. Juxta fused to phallus, with elongate, flattened ventral process connecting phallus-juxta complex to base of valves. Phallus very elongated, curved, downturned, terminus with hook-like backwardly-angled process, base of phallus with elongated lobe. **Female.** Unknown.

#### Distribution

(Fig. 19). *Auroriana colombiana* is so far known only from the type locality in central Colombia.

#### Etymology.

This species is named for Colombia, as it is the only species in the genus known from this country.

#### Remarks.

Although this species resembles *Auroriana florianensis*, the striking genital differences clearly differentiate the two. *Auroriana florianensis* and *Auroriana colombiana* seem to represent an Amazonian lineage of *Auroriana*, unified by the presence of heavier setae on the saccular basal edge of the valves and by the relatively large, elongate phallus, characters that are not present in *Auroriana gemma* sp. n. described below.

### Auroriana florianensis

Taxon classificationAnimaliaLepidopteraMimallonidae

(Herbin, 2012) comb. n.

**Figures 15, 16.**
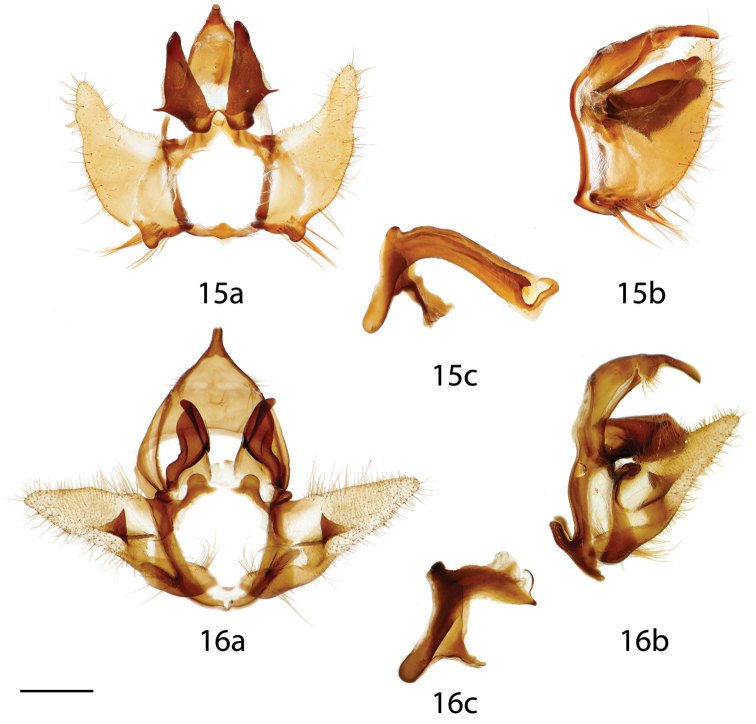
*Auroriana* male genitalia, **a** ventral, **b** lateral, **c** phallus. **15** *Auroriana florianensis*, French Guiana, Massif du Mitaraka, 300 m [St. Laurent diss.: 11-19-15:1] (RAS) **16** *Auroriana gemma* holotype, Brazil, Santa Catarina, São Bento do Sul, Rio Natal, 550 m [C. Mielke diss.: 27.473] (DZUP). Scale bar = 1 mm.

**Figure 17.**
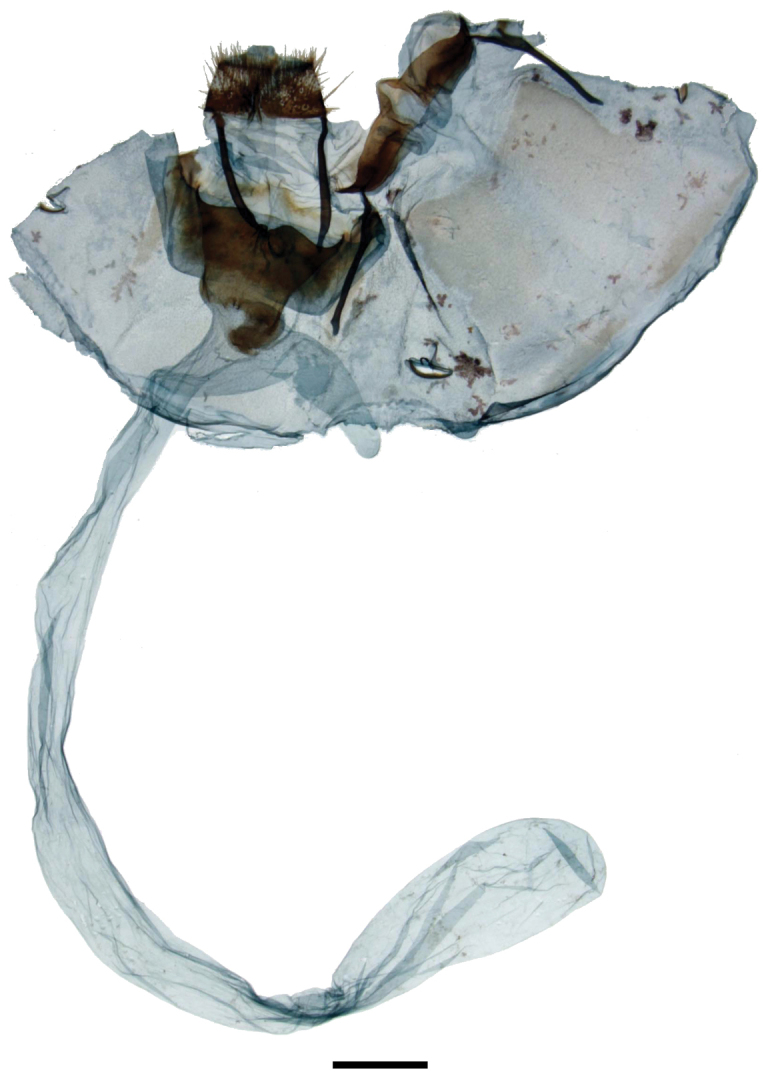
*Auroriana florianensis* female genitalia. French Guiana, Réserve des Nouragues, Inselberg, 110 m [C. Gibeaux diss. prep: 7759] (MNHN). Photo courtesy of C. Gibeaux. Scale bar = 1 mm.

**Figure 18.**
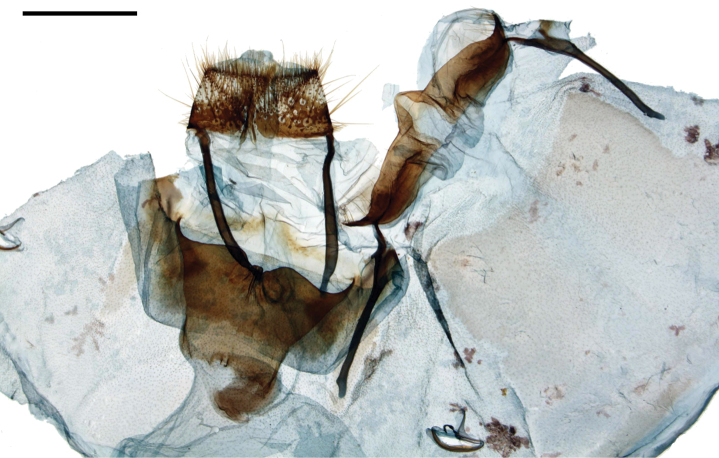
*Auroriana florianensis* female genitalia, detail. As for Figure 17. Scale bar = 1 mm.

**Figure 19.**
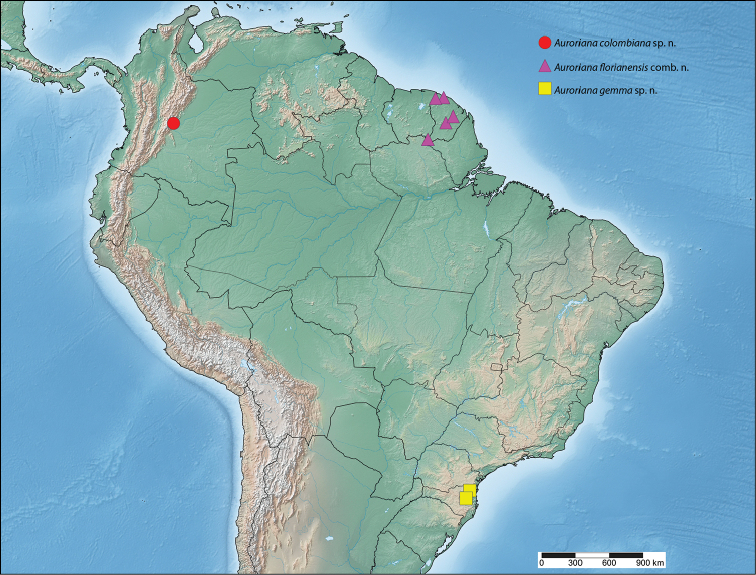
Distribution of *Auroriana*.

Figs 9–11 , 15 , 17 , 18 , 19

Cicinnus florianensis Herbin, 2012: 23–25, figs 17, 18.

#### Type material.

**Holotype**, ♂: 29-VII-2001, Piste du Dégrad Florian, PK 12, GUYANE Fcse, Coll. M. LAGUERRE, M. Laguerre leg./ genitalia, prep. D. Herbin, H. 674/ BC-Her1657/ Cicinnus florianensis, Herbin, 2012, The European entomologist, Vol. 4, N° 1, Holotype ♂/ (MNHN, examined). Type locality: French Guiana: Piste du Dégrad Florian, PK 12.

#### Additional specimens examined.

(4 ♂, 1 ♀) **FRENCH GUIANA**: 1 ♂, St. Jean du Maroni, Plateau des Mines, PK 1,3: 29.VII.1993, L. Sènécaux réc. (MNHN). 1 ♂, Mont Mitaraka [Massif du Mitaraka], 300 m: 19.VIII.2015, La Planète revisitée, MNHN/PNI, APA 973-I, Ph. Collet leg., at mercury vapor light, St. Laurent diss.: 11-19-15:1 (RAS). 1 ♂, Saül, Point de vue: 30.VII.2011, Eddy Poirier leg., at UV light (CPC). 1 ♂, Réserve des Nouragues, Inselberg: 5.VIII.2010, Ph. Collet leg., at UV light (CPC). 1 ♀, Réserve des Nouragues, Inselberg, 4° 5’15” N, 52° 40’48” W, 110 m: 8.VIII.2010, at UV light, Eddy Poirier leg., Chr. Gibeaux diss. prep: 7759 (MNHN).

#### Diagnosis.

*Auroriana florianensis* is similar to the previous species; see the diagnosis of *Auroriana colombiana* for differentiating characters between both species. *Auroriana florianensis* can be distinguished from *Auroriana gemma* sp. n. by the more strongly curved forewing postmedial line, straighter forewing antemedial line, and by the more expansive pink suffusion in the postmedial region. The genitalia can also be used to differentiate the two: in *Auroriana florianensis* the tegumen is narrower, the gnathos processes larger, with a sharp tooth basally, and the valves are bent upward, with heavier setae on the basal saccular edge. *Auroriana florianensis* lacks the mesal valve tooth present in *Auroriana gemma* sp. n. Finally, the phallus of *Auroriana florianensis* is much longer than in *Auroriana gemma* sp. n.

#### Description.

**Male.** *Head*: Pink, eyes bordered posteriorly by dark-brown scales; antenna opaque yellow; labial palpus small, dorsally with darker scales. *Thorax*: Pink, interspersed with beige scales, prothorax darker pink. *Legs*: Light pink, vestiture long, tarsus beige, tarsal spurs relatively long, finely pointed apical tips. *Forewing dorsum*: Forewing length: 17 mm, avg. 17 mm, n = 3, Wingspan: 32 mm. Triangular, slight inward notch at tornus, margin convex mesally. Ground color light orange brown, overall very sparsely speckled by dark petiolate scales. Antemedial line faint pink, nearly straight. Postmedial line bowed inward, dark brown, outward edge lined with pale pink, especially approaching tornus, postmedial line angled sharply towards costa immediately after passing Rs4. Antemedial area pink. Medial area lighter orange with pink suffusion. Submarginal area darker brown, marginal area with variably sized pink-gray suffusion. Discal spot a small black mark. *Forewing ventrum*: Similar to dorsum but ante- and postmedial lines absent, pink suffusion reduced antemedially and medially, discal mark more visible. *Hindwing dorsum*: Coloration as for forewing dorsum, but less pink; postmedial line lacks outer pink edging. *Hindwing ventrum*: Follows same pattern as forewing ventrum, but postmedial line diffuse, brown, outwardly lobed mesally. *Abdomen*: As for genus but coloration more beige than pink. *Genitalia*: (Fig. 15) n = 1. Tegumen broad, subtriangular. Vinculum somewhat rectangular. Uncus moderate length, tubular, hardly differentiated from tegumen. Gnathos as two unfused, heavily-sclerotized, somewhat triangular processes with sharp basal tooth. Simple triangular valves bent upward, narrowed distally. Base of valves with heavier, differentiated setae, heavily sclerotized lobes. Juxta fused to phallus, with wide ventral process connecting phallus to base of vinculum. Phallus elongated, curved, downturned, terminus with weak hook-like curl and heavily sclerotized forward pointing process. Base of phallus with elongated lobe. Vesica thick, bag-like. **Female.** *Head*: As for male, but antennal rami shorter. *Thorax*: As for male, but with more beige scales. *Legs*: As for male. *Forewing dorsum*: Forewing length: 17 mm, Wingspan: ~33.5 mm, n = 1. As for male, but broader, pink suffusions reduced, ground color more olive green than orange [possible artifact of photography]. *Forewing ventrum*: As for forewing dorsum but pinkish suffusions nearly absent, postmedial line absent, discal mark more pronounced. *Hindwing dorsum*: As for male but slightly broader, dark petiolate scales more numerous, especially antemedially. *Hindwing ventrum*: Following same pattern as forewing ventrum. *Abdomen*: As for male, but slightly broader. Sternite VIII with pair of short sclerotized bands posteriorly. *Genitalia*: (Figs 17, 18), n = 1. Papillae anales broad, somewhat rectangular, covered in setae, longer anteriorly. Apophyses anteriores with curved tips; apophyses posteriores robust, wide, slightly longer and broader than apophyses anteriores. Ductus bursae very long, somewhat sclerotized near ostium, ductus not differentiated from long, narrow corpus bursae. Appendix bursae present, elongate, fingerlike. Tergite VIII as narrow band with posteriorly directed mesal arc with membranous center. Lamella antevaginalis very large, broad, trapezoidal, notched mesally.

#### Distribution

(Fig. 19). *Auroriana florianensis* is so far known only from French Guiana, but is widely distributed in the region.

#### Remarks.

At the time of its original description, *Auroriana florianensis* was first suggested by Herbin (2012) to be unique among Mimallonidae and possibly belonging to a new genus. Despite this opinion, with which we agree, Herbin placed this species in the “catch-all” genus *Cicinnus*, which is currently being used to subsume many newly described taxa that show no obvious generic associations (Herbin 2012, 2015, Herbin and Mielke 2014, Herbin and Monzón 2015).

After reading the description of *Cicinnus florianensis*, the first author recognized that two undescribed species under study were congeneric with *Cicinnus florianensis*, but none of them belonged in *Cicinnus*. We further support Herbin’s (2012) original assertion that *Cicinnus florianensis* belonged to a new genus. This realization was solidified when genitalia dissections revealed that the characters previously thought wholly unique to *Auroriana florianensis* were indeed shared with both *Auroriana gemma* sp. n. and *Auroriana colombiana*, and with no other described Mimallonidae.

Until now, *Auroriana florianensis* was only known from the holotype male. The female and its genitalia are figured for the first time. Additional distributional and temporal data are presented for this species.

The apparent rarity of *Auroriana florianensis* is noteworthy, especially considering that this species is known from only five males and one female, despite intensive collecting efforts in French Guiana. All specimens were collected in the dry season, the last days of July until late August, displaying a rather restricted flight period of less than a month. However, there are less collecting efforts during the dry season (C. G. Mielke pers. obs.), thus it is not possible to determine if the few records are due to this season being poorly collected or true rarity of the species.

### Auroriana gemma

Taxon classificationAnimaliaLepidopteraMimallonidae

St Laurent & C. Mielke sp. n.

http://zoobank.org/CFB7093A-C529-439F-867C-3756B16ACB46

Figs 12 , 16 , 19

#### Type material.

**Holotype**, ♂: BRAZIL – SC [Santa Catarina], São Bento do Sul, Rio Natal, 550 m. XI.2013. A. Rank leg/ 27.473 Col. C. Mielke / C. Mielke diss.: 27.473/ DZ 32.729/ HOLOTYPE male *Auroriana gemma* St Laurent and C. Mielke, 2016 [handwritten red label]/ (DZUP). Type locality: Brazil: Santa Catarina: São Bento do Sul, Rio Natal.

**Paratype**, 1 ♂: **BRAZIL: Santa Catarina**: Neu Bremen [Dalbérgia]: III.1938, B. Pohl (MZSP). Paratype with the following yellow label: PARATYPE male *Auroriana gemma* St Laurent and C. Mielke, 2016.

#### Diagnosis.

*Auroriana gemma* can be easily distinguished from its two congeners by the reduction of the pinkish suffusion postmedially and by the lighter medial and darker postmedial regions. Additionally, the forewing postmedial line is outwardly lined with a thicker pale-pink band than in other species in the genus. The hindwing postmedial line in *Auroriana gemma* is diffuse rather than thin and dark as in the other two species. The male genitalia also readily differentiate this species from the others. In *Auroriana gemma* the tegumen is broader, gnathos processes shorter, somewhat wrinkled, and without a sharp tooth proximally. Additionally, the valves bear a unique, distinct, triangular mesal tooth and lack differentiated setae at the base of the valves. Finally, the phallus of *Auroriana gemma* is much shorter than in either of the other *Auroriana* species.

#### Description.

**Male.** *Head*: Light pink, eyes bordered posteriorly by dark scales; antenna opaque yellow. *Thorax*: Light tan brown, interspersed with pink scales, especially near wing base, prothorax pink. *Legs*: Light pink, but tarsus light brown. *Forewing dorsum*: Forewing length: 16 mm, avg. 16 mm, Wingspan: 30 mm, n = 2. Short, triangular, inward notch at tornus reduced, margin convex mesally. Ground color amber, orange brown, overall lightly speckled by dark petiolate scales. Antemedial line very faint pink. Postmedial line slightly curved, gray, outward edge lined with very pale pink, postmedial line angled sharply towards costa immediately after passing Rs4, becoming diffuse pink suffusion. Antemedial area pink. Medial area lighter orange with pink suffusion. Postmedial area darker brown with pale pink-gray suffusion near wing margin; medial area lighter orange with pink suffusion; antemedial area pink. Discal spot a small dark-gray line across width of discal cell. *Forewing ventrum*: Similar to dorsum but with more speckling; ante- and postmedial lines absent; deeper orange overall with more expansive, defined pink suffusion postmedially. *Hindwing dorsum*: Coloration as for forewing dorsum, but less pink; postmedial line only as pale pink suffusion. *Hindwing ventrum*: Follows same pattern as forewing ventrum but more pink overall, orange coloration restricted to marginal region. *Abdomen*: As for genus, coloration as for thorax but more golden beige dorsally, pinker ventrally. *Genitalia*: (Fig. 16) n = 2. Tegumen broad, subtriangular. Vinculum irregular with paired, anterior process, rounded lobes present mesally below gnathos. Uncus short, tubular, hardly differentiated from tegumen. Gnathos as two unfused, heavily sclerotized, wrinkled, somewhat triangular processes. Triangular valves small relative to tegumen, simple, with sharp triangular mesal tooth. Juxta fused to phallus, with acute, pointed process. Phallus very short, simple, downturned, pointed with thin, sclerotized accessory extension terminally. Vesica short, bag-like, bulbous ejaculatoris twice length of phallus, bag-like. **Female.** Unknown.

#### Distribution

(Fig. 19). *Auroriana gemma* is known from only two specimens: one at the type locality at 550 m in northeastern Santa Catarina state, Brazil, and the other from about 80 km farther south in the same state.

#### Etymology.

This species is named for the amber (*gemma* Latin) ground color. The name is doubly appropriate because *gemma* also translates to gem, which refers to the beauty and rarity of this species.

#### Remarks.

This new species is known from only two specimens, both of which surprisingly come from very well collected regions of southeastern Brazil (R. A. St. Laurent & C. G. Mielke pers. obs.). Given that one specimen was collected in March and the other in November, we cannot consider a short flight period as the reason for the apparent rarity of this species, as we mentioned for *Auroriana florianensis*. All *Auroriana* species are known from very few specimens, suggesting that *Auroriana* in general is not an easily collected genus or one that is merely overlooked by lepidopterists, as is much of the family.

### Micrallo

Taxon classificationAnimaliaLepidopteraMimallonidae

St Laurent & C. Mielke gen. n.

http://zoobank.org/56E27AA5-B571-4CB7-8223-1DCD3AC7E381

#### Type species.

*Micrallo minutus* St Laurent & C. Mielke, sp. n.

#### Etymology.

The genus name is based on the fact that the type species is remarkably small (*micro*- Latin), being one of the smallest in the family; + the ending –llo, which is shared with *Mimallo* Hübner, [1820], the type genus of Mimallonidae. The genus name is masculine.

#### Diagnosis.

*Micrallo minutus* is immediately distinguished from all described Mimallonidae by the small size (forewing length: 11.5 mm) combined with the silvery-gray ground color and smooth wing margins. Genitalia are also unique in both sexes. Males have extremely complex genitalia with semi-membranous valves and lateral pockets encasing the valves. The pockets are filled with thick, elongate deciduous setae. Female genitalia have bent apophyses anteriores and long, robust apophyses posteriores that are connected to paired pouches that open on either side of the papillae anales. In the single examined female, one (the left hand pouch when viewed ventrally) of the two pouches was completely filled with elongate, thick setae that appear to originate from the male. *Cicinnus acuta* (Schaus, 1892) is superficially similar, but much larger and lacks the club-like valves and differentiated setae in lateral pouches of the male genitalia.

#### Description.

**Male.** *Head*: Width roughly two thirds that of thorax, antenna bipectinate to tip; basal three rami pairs increasing in length from antennal base to roughly one fifth of antennal length where rami length is nearly constant for remainder of antenna until the final distal quarter, after which rami continuously decrease in length until terminus. Frons tan brown, labial palpus reduced, not extending beyond frons, second segment tufted, third segment highly reduced, indistinct. *Thorax*: Light gray, interspersed with darker petiolate scales, especially anteriorly. *Legs*: Gray brown, but tarsus darker brown, tibial spurs thin. *Forewing dorsum*: Forewing length: 11.5 mm, Wingspan: 23 mm, n = 1. Somewhat narrow, acutely triangular, apical third slightly concave, somewhat convex before tornus. Ground color light silvery gray, overall lightly speckled by dark petiolate scales. Antemedial line absent. Postmedial line light brown, angled outward from M_3_ to Rs3, angled sharply towards costa immediately after passing Rs4, line straight from M_3_ until posterior wing margin, outer edge of postmedial line lined with pale coloration, becoming darker at costa. Postmedial area darker graphite colored with pale pink-gray suffusion mesally. Discal mark black, oblique, somewhat ovoid in shape. *Forewing ventrum*: Similar to dorsum but browner medially, especially on costa; inner side of postmedial line with black suffusion, postmedial line more undulate than on dorsum. *Hindwing dorsum*: Coloration as for forewing dorsum, but browner, especially anteriorly; postmedial line more undulate than on forewing. *Hindwing ventrum*: Follows same pattern as forewing ventrum. Frenulum apparently absent or vestigial. *Venation*: Typical of Mimallonidae, very similar to *Cicinnus melsheimeri* but Rs3 + Rs4 much longer stalked. *Abdomen*: Short, extending slightly beyond hindwing tornus, almost tubular, coloration as for thorax but lighter gray ventrally. *Genitalia*: (Fig. 22), n = 1. Extremely complex; tegumen reduced, narrow, but with robust sclerotized margins. Vinculum circular, but heavily modified, variously connected to valves; pair of heavily sclerotized, curved, tusk-like structures originate near base of vinculum. Uncus reduced, bottle shaped. Gnathos as two unfused, outwardly sclerotized, inwardly membranous, elongated, tubular, mesally bent processes with two pairs of teeth mesally, two pairs basally. Valves highly modified, nearly absent, valves membranous proximal to vinculum, transitioning into club-shaped lobes distally, lobes extend nearly to apex of uncus. Base of valve with elongate, thick, deciduous, specialized setae, most of which contained in membranous fold laterally encasing valve-vinculum complex. Juxta partially fused to phallus, juxtal plate a reduced, roughly T-shaped structure embedded in anellus. Phallus simple, spade shaped (viewed dorsally), open dorsally, base with elongated, downward-angled process. **Female.** *Head*: As for male but antenna smaller overall with shorter rami. *Thorax*: As for male. *Legs*: As for male. *Forewing dorsum*: Forewing length: 11.5 mm, Wingspan: 22 mm, n = 1. As for male but browner, discal spot absent. *Forewing ventrum*: Similar to dorsum but browner, especially anteriorly and medially; inner side of postmedial line with black suffusion, postmedial line more undulate than on dorsum. *Hindwing dorsum*: As for male but browner. *Hindwing ventrum*: Follows same pattern as forewing ventrum. Frenulum apparently absent or vestigial. *Abdomen*: As for male but slightly more robust; sternite VIII with quadrate U-shape formed by thin sclerotized band spanning width and length of segment. *Genitalia*: (Figs 23, 24), n = 1. Papillae anales small, rounded, covered in fine setae. Apophyses anteriores thin, distal third angled dorsally; apophyses posteriores, robust, nearly straight, twice length and width of apophyses anteriores. Ductus bursae same length as segment VIII, ostium unsclerotized. Corpus bursae bag-like, without any sclerotized structures. Dorsal sclerotization of tergite VIII complex, ridged, forming posteriorly directed point. Lamella antevaginalis a very large plate, nearly of equal width as segment VIII, with deep, wide, mesal indentation forming ostium. Pair of specialized, lateral pouches on either side of papillae anales, pouches connected to apophyses posteriores. Viewed ventrally, right pouch filled with elongated, deciduous setae, left pouch empty.

**Figure 20, 21.**
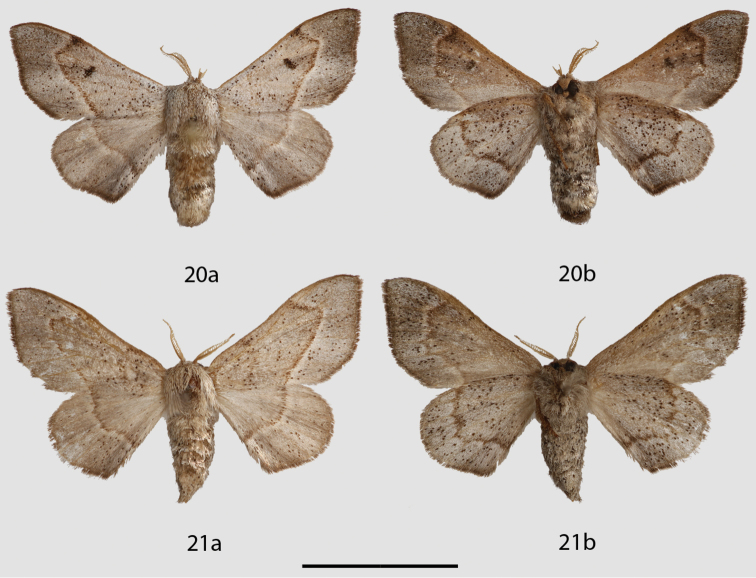
*Micrallo minutus* adults, **a** dorsal, **b** ventral. **20** Holotype ♂, Brazil, Piauí, Oeiras, 200 m (DZUP) **21** Paratype ♀, Brazil, Piauí, Oeiras, 200 m (USNM). Scale bar = 1 cm.

**Figure 22.**
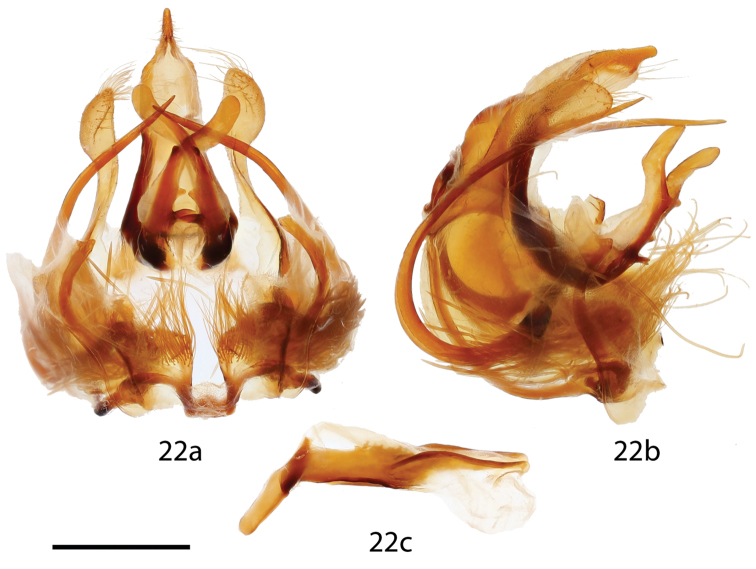
*Micrallo minutus* male genitalia, **a** ventral, **b** lateral, **c** phallus. Holotype ♂, Brazil, Piauí, Oeiras, 200 m [St. Laurent diss.: 10-21-15:1] (DZUP). Scale bar = 1 mm.

**Figure 23, 24.**
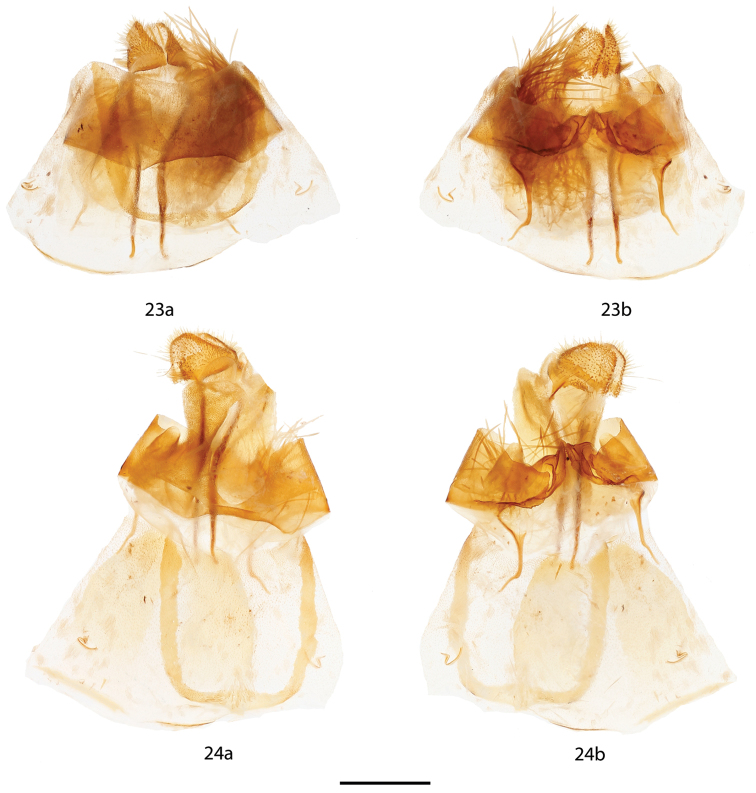
*Micrallo minutus* female genitalia, **a** ventral, **b** dorsal. **23** Paratype ♀, Brazil, Piauí, Oeiras, 200 m, before extension [St. Laurent diss.: 10-21-15:2] (USNM) **24** As for Figure 23, after extension. Scale bar = 1 mm.

#### Remarks.

An examination of *Cicinnus acuta* genitalia preparations (Franclemont genitalia prep. 1768 (CUIC) and St. Laurent diss.: 10-25-15:1) reveals minor similarities in the shape of the phallus, wherein both species the phallus is rather flattened and spade shaped when viewed dorsally. *Cicinnus acuta* differs, however, in all other respects of genitalia structure, namely in the presence of well-defined valves, much shorter gnathos processes, and in the presence of sharp, almost pincer-like structures at the base of the valves.

### Micrallo minutus

Taxon classificationAnimaliaLepidopteraMimallonidae

St Laurent & C. Mielke sp. n.

http://zoobank.org/B04B1CB2-8C94-4323-AD47-13A6A4404975

**Figure 25.**
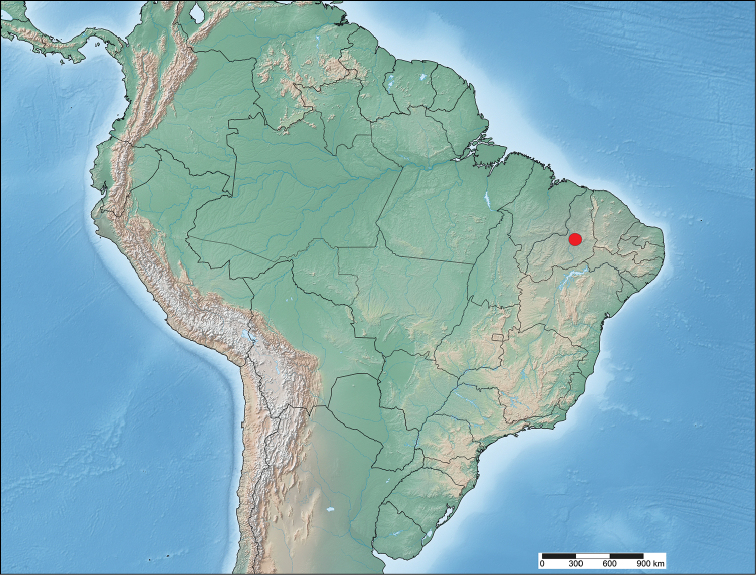
Distribution of *Micrallo minutus*.

Figs 20–21 , 22 , 23, 24 , 25

#### Type material.

**Holotype**, ♂: BRASIL: PI [Piauí], Oeiras. 200 m, 12.iv.1994, V.O. Becker Col/ Col. Becker 92248/ USNM-Mimal: 2376/ St. Laurent diss.: 10-21-15:1/ DZ 32.730/ HOLOTYPE male *Micrallo minutus* St Laurent and C. Mielke, 2016 [handwritten red label]/ (ex. USNM, to be deposited in DZUP). Type locality: Brazil: Piauí: Oeiras.

**Paratype**, 1 ♀: **BRAZIL: Piauí**: Oeiras, 200 m: 12.IV.1994, V.O. Becker Col., Col. Becker 92248, USNM-Mimal: 2377, St. Laurent diss.: 10-21-15:2 (USNM). Paratype with the following yellow label: PARATYPE female *Micrallo minutus* St Laurent and C. Mielke, 2016.

#### Diagnosis.

See genus diagnosis.

#### Description.

See genus description.

#### Distribution

(Fig. 25). The unique species in the genus *Micrallo* is so far known only from the type locality at Oeiras, Piauí, Brazil. This location is interesting because it lies on the edge of both Cerrado and Caatinga biomes (IBGE 2004).

#### Etymology.

*Micrallo minutus* is named for its minute (*minutus* Latin) size, making it one of the smallest described species in the family.

#### Remarks.

Due to the interesting habitat at the type locality of *Micrallo minutus*, it is not surprising that this taxon represents a previously undescribed species in a new genus. Both Caatinga and Cerrado biomes are incredibly undersampled (C. G. Mielke & R. A. St. Laurent pers. obs.), with the latter biome recently proven to support many new and endemic Mimallonidae species (Herbin and Mielke 2014). As of yet, no study has been published to determine the degree of Mimallonidae endemism to Caatinga.

The highly specialized genitalia in both sexes are unique among Mimallonidae. The complicated male genitalia bear two pouches on either side of the semi-membranous valves that contain many thick, specialized setae that are prone to falling out when the genitalia are examined. The female, likewise has rather complicated genitalia for female Mimallonidae, and also has specialized pouches, one on each lateral side of the papillae anales. In the single available female specimen, the right (when viewed ventrally) pouch contained an abundance of the same setae, the pouch was so completely filled that many of the setae are extending outside the pouch. See Fig. 23 for the genitalia before extension of the final abdominal segment. After extension (Fig. 24), most of the setae were expelled from the pouch. Close examination shows that the female genitalia are not asymmetrical, but that the second pouch was simply never filled with setae, or if it did contain setae at one time, they were completely lost before our examination. It is possible that these setae have characteristics pertinent to copulation, although we are unable to determine their exact purpose. It is interesting that only one pouch was filled, suggesting that females of *Micrallo minutus* are capable of mating two or more times. Perhaps even more intriguing is the fact that males may be able to fill only one female pouch at a time, despite possessing two complimentary pouches of their own on either side of the genital capsule.

## Supplementary Material

XML Treatment for Tostallo

XML Treatment for Tostallo albescens

XML Treatment for Auroriana

XML Treatment for Auroriana colombiana

XML Treatment for Auroriana florianensis

XML Treatment for Auroriana gemma

XML Treatment for Micrallo

XML Treatment for Micrallo minutus
